# Cancer complexity: why we need a novel cancer research strategy

**DOI:** 10.3389/fonc.2025.1624467

**Published:** 2025-08-01

**Authors:** Anders Bredberg

**Affiliations:** ^1^ Department of Laboratory Medicine, Lund University, Lund, Sweden; ^2^ Department of Medical Microbiology, Innlandet Hospital Trust, Lillehammer, Norway

**Keywords:** cancer complexity, chaos theory, tinkering, cancer treatment, research strategies

## Introduction

There seems to be a growing consensus that complexity is an overarching feature of cancer (1–4). At the same time there is confusion regarding the meaning of the terms ‘complexity’ and ‘complex’, which presumably denote two linguistic forms of the same thing: if something is complex then it displays complexity. Some scientists seem to think about the growing number of molecules (or ‘targets’) which are found to play a role for cancer, while others emphasize influences from the whole body with its many organs, metabolism, obesity, microbial floras, or influences from environmental factors such as diet, smoking and radiation (5–13). However, despite this confusion, most of cancer reports from the last few years appear to use complexity to describe how cancer researchers and physicians are becoming overwhelmed by the tsunami of new data revealing a perplexing multifaceted picture.

This opinion article argues that cancer complexity represents a pathogenetic mechanism, rather than simply a large number of ‘hallmark’ molecular aberrations grouped into a collection of ‘clouds’ (such as obesity and neural-cancer crosstalk) (1). Furthermore, it outlines how today’s targeting-based cancer research can be supplemented with a novel strategy type. Cancer as we know it today has much in common with a description of complexity emanating from the physical sciences. Nevertheless, cancer is often addressed in the clinic with a problem-solving targeting strategy known to work well with complicated problems. This distinction between complicated and complex is important because, when it is viewed together with two general observations outlined in this commentary, then it implies that the currently dominating cancer research strategy needs to be supplemented with a novel strategy.

## The clinical benefit coming from modern cancer research

The first general observation is about the impact cancer research has had on patients. The technically advanced research of today lags the old workhorses chemotherapy and radiation as far as therapeutic benefit is concerned. Cytotoxic drugs, originally derived from mustard gas, still brings the cure to more patients than modern targeting agents (14). This is most evident for leukemia, lymphoma (with the CHOP drug cocktail estimated to have cured 20,000 DLBCL patients annually in the USA for the last 30 years) and testicular cancer. Perhaps the most notable therapeutic advance of all times has been the cure of childhood leukemia by the ‘Total Therapy’ cytotoxic protocols designed in the 1960s and 1970s (15).

However, the way modern molecular science helps many patients to survive much longer thanks to targeting drugs like monoclonal antibodies and small molecule kinase inhibitors must not be under-appreciated, and there have been spectacular advances in melanoma and hematological malignancies. There are many examples of how multi-omics data has benefitted clinical cancer care by reducing recurrence and increasing long-term survival. These data include spatial transcriptomics and a dynamic regulatory network mapping of a tumor stress response to oxidative damage (16–18). Large studies on early stage HER2-positive breast cancer have shown that adding an anti-HER2 monoclonal antibody to chemotherapy reduces 10-year mortality by a third from 21.1% to 14.7% and correspondingly the recurrence rate from 31.9% to 22.9% (19), and the same research team reported later that while a long-term risk of distant recurrence remains, it is about a tenth lower in the time period after anti-HER2 therapy was introduced (20). Five-year mortality in 500,000 women with early invasive breast cancer has gradually decreased over the period 1990 to 2015 from 14.4% to 4.9%, and the authors state that while most of this effect is due to surgery, adjuvant therapies such as chemotherapy, hormones and targeting agents will contribute to the reduced long-term mortality (21). It has furthermore been reported that genomic medicine in combination with multi-omics has transformed breast cancer care; e.g., with poly (ADP-ribose) polymerase (PARP) inhibitors effectively reducing recurrence risk in BRCA-mutated patients; and with several prognostic biomarkers and precision treatment regimens now being under development (22). There are similar results for lung cancer documenting a significant clinical benefit of modern targeting treatment. Two-year survival improved from 26% in men diagnosed in 2001 to correspondingly 35% in 2024; since targeting modalities, especially for the EGFR-positive NSCLC lung cancer subtype, was introduced in 2013 this improvement is arguably linked to this addition to chemotherapy (23). The remarkable success with a curative effect from immune checkpoint blockade (ICB) in circa 50% of patients is still largely limited to patients with malignant melanoma, but response rates of up to 25% has been attained in some other forms of solid cancer, and combinations with non-targeting agents as well as development of drug conjugates featuring an ICB component show great promise (24, 25). Nevertheless, it should be realized that there is often a relatively modest clinical benefit coming from addition of a targeting drug to conventional treatment, and this can be illustrated by an example. The FDA (US Food and Drug Administration) in 2024 approved the addition of an immune checkpoint blockade PD-1 inhibitory monoclonal antibody to standard chemotherapy for advanced mesothelioma (NCT02784171). The main measure in this trial was overall survival, where the median became significantly (p=0.0162) improved to 17.2 months as compared with 16.1 months for the standard regimen, and progression-free survival was 7.1 months in both groups.

To summarize this first general observation, while 50 years of molecular dissection of cancer has greatly benefitted cancer patients by prolonging survival, there is reason to doubt that it will have a major curative impact for patients within a reasonable timeframe.

## Complexity of both cancer and normal physiology

The second general observation is about the true scale of the quantity of factors that are involved in cancer, and about how normal physiology controls many of these factors (26, 27). The devil is sometimes in the details: just consider the path leading up to mutations: at least a thousand DNA bases become spontaneously degraded in each human cell each day and form potentially mutagenic lesions. This damage initiates the physiologic concerted action of >100 DNA repair enzymes and many of the >500 human kinases. Furthermore, consider that 3 mutations, on average, are formed, despite all DNA damage response activities, each of the 10^16^ times that the 3x10^9^ DNA base pairs/cell are duplicated during a human (with >10^13^ cells) lifetime.

One aspect of this physiology is the maintenance that keeps the whole body fit in the face of the myriad threats encountered by virtually every cell every day. An important question is whether the aim of this defense is maximal elimination of the threat or if instead defense is regulated and seeks some kind of optimum. The presence of stimulating and inhibitory activities searching for an optimum balance point suggests that the answer to this question is that physiologic defense is not striving for maximal effect. It is not only made up of very many molecular factors, but in addition each defense mission is regulated to reach a precise point of balance between, on the one hand, activities directed against the threat and, on the other, opposing activities mediating feed-back inhibition of this attack. This delicate regulation ensures acceptable levels of benefits resulting from neutralization of the threat and costs in the form of collateral damage to healthy tissues and energy consumption. The regulation is most probably dynamic in the sense that one and the same threat, when encountered repeatedly at different times by one and the same individual, leads to a spectrum of different balance points, being seemingly randomly distributed. Thus, this physiologic defense may very well be judged to display complexity.

Physiologic defense against cancer is executed on many frontiers. The extent to which the large quantity of DNA damage can cause cancer-associated mutations is controlled on the DNA damage response frontier. This frontier is no less essential than the immune system for safeguarding us against cancer. It reduces the formation of mutations but comes with a cost when it acts off-target and mediates chromosome translocations causing lymphoma and some epithelial cancers. Interestingly, one player on this frontier, the V(D)J recombinase enzyme complex, is also part of the immune system. On this second frontier, the immune system regulates and drastically reduces the extent to which precancerous mutated cells (which have slipped through the DNA damage response safety net) will progress all the way into a clinical tumor. Here, a balance point with relatively *little* of attack on these cells is coupled to a risk for a clinically overt case of cancer, while a balance point with relatively *much* of attack comes with a cost in the form of autoimmunity. Additional evidence that the efficiency of defense against cancer is regulated is provided by a much-debated paradox (Peto’s from 1975) (28–30). The paradox lies in the observation that while a single mutated cell is the origin of each cancer, there is a similar cancer frequency among mammals with different total cell number, ranging from a whale composed of 10^17^ cells to a bumble bee bat with only 10^9^ cells.

While the balance ‘optimum’ and ‘acceptable’ points within the physiologic defense against threats presumably have been fixed during evolution to serve the fitness of the human species, they will not necessarily function to keep the human individual healthy. For example, it would be desirable for an individual to have a more efficient (perhaps on par with the whale) defense against tumors. Conversely, one reason for evolution to permit an acceptable level of cancer may be to allocate energy to other tasks than a more efficient DNA damage response.

To summarize this second general observation, it shows that the concept of physiologic regulatory processes with dynamic balance points adds a new layer to cancer complexity.

These processes can be divided into three phases:

- a vast quantity of molecular factors contributes to cancer,
- normal physiology will process these factors so that their contribution to cancer becomes reduced and reaches an ‘optimum’ ensuring that
- eventually an ‘acceptable’ level of cancer disease is reached.

## Implications for the future of cancer research

The two outlined general observations indicate that cancer has much in common with how complexity is viewed within some branches of the physical sciences (31–39). A complicated system or problem like a human-made machine typically contains many components, with well-known properties and interactions. Stimulating or blocking a component, being the principle of drugs targeting a specific molecule, has a predictable and reproducible effect. If cancer were such a problem, the current cancer research strategy would be ideal. In contrast, a complex problem may involve unknown or weakly defined parts and is ‘dynamic’ (meaning that it is continuously modified as both time and its multi-dimensional organization change). Furthermore, it is difficult to predict the effect resulting from manipulation of a specific component. If we find that our present view on cancer fits with this description of complexity, then it is not surprising that targeting a single or a few molecules will seldom lead to a cure, or that a treatment’s benefit and side-effects cannot be predicted in each patient.

It is reasonable to doubt that we can expect radically increased cure rates from a continued mapping strategy the way it has recently, in all its essence, been outlined as our way forward to embrace cancer complexity (1, 4, 40–42). Previous technological revolutions, like recombinant DNA revealing oncogenes and the mapping of the human whole genome sequence, could not meet the initial great expectations to win the ‘war on cancer’. Systems biology is based on the assumption that the whole of complexity is more than the sum of its components, and that it includes also interactions and dynamic alterations in space and time of components. Although the omics revolution doubtless will give us a deeper understanding of cancer, it seems just possible that the real-life complexity of cancer is far too vast for even this human achievement to be able to provide the cancer cure.

We should therefore plan for a supplementary type of strategy which is not built solely on modeling of discrete molecular data, but rethink cancer management as dynamic rather than static, and take advantage of experiences coming from chaos theory and from a trial-and-error approach often referred to as tinkering. Recent developments in systems biology computational modeling of multi-omics data have provided significant information on the dynamics of molecular aberrations in cancer and on predictability of how they develop over time and in space. There are biological phenomena which, in addition to randomly distributed events, display so-called chaotic large-scale behavior (28–32). This behavior follows simple rules, which allow for predictability of the effects resulting from a manipulation. One example of such chaotic behavior is ‘patterns’, both in morphology as well as in dynamic circadian oscillations of for example intracellular calcium and transcription factor concentrations, cell cycle control and tumor-immune interactions (18, 29–35, 43). Systems biology modeling approaches constitute an attempt to address the basic problem of predictability inherent in complex systems. It should be acknowledged that as yet such approaches have mainly been preclinical and conceptual in character, and have played a clearly minor role for practical clinical recommendations. However, there are some recent reports on models touching on chaotic behavior which have provided robust actionable tools (34, 36, 44–46). A glioblastoma – immune system network study identified possibly clinically targetable so-called bifurcation dynamics within tumor phenotypic transitions, suggesting a causal pattern driving tumor evolution and cell fate decisions (36). The result was suggested to provide proof of concept evidence that such a complex pattern can assist clinical therapeutic decisions. A multicenter clinical pathology diagnostic work using multi-omics data demonstrated that advanced mathematic modeling can ‘deconvolute’ the massive data quantity and thereby reveal complex patterns of tumor microenvironment dynamic organization, which provide significant information guiding clinical therapeutic decisions (46). Finally, another study touching on chaotic behavior applied a complexity type of approach named ladderpath (describing how a problem can be decompressed into hierarchical structures using repetitive elements) to spatial multidimensional dynamical systems (47). Interestingly, it is discussed in this report how the ladderpath approach is grounded not only in Kolmogorov complexity computation, but was also inspired by a tinkering process being suggested by the assembly theory on evolutionary biology (48), where reuse of existing molecular components is viewed as being central in the dynamics of complexity. Furthermore, the authors of the ladderpath study (47) relate how this idea on reuse as a mechanism within complex systems to generate new components and interactions was conceived by François Jacob (49) and named evolution by tinkering. They also relate how this tinkering concept still today leads to new research with a fresh perspective and which shows potential in fields such as drug discovery.

Indeed, as already mentioned, the cure of childhood leukemia (15) was accomplished with this tinkering type of strategy (50–53). A panel of pediatricians then assembled to formulate a qualified guess on how to maximize the benefit of a reuse of all, at that time, available cytotoxic agents without getting a fatal cost. This landmark discovery was not inspired by results from molecular mapping experiments. The therapy causes cytotoxic damage to an infinite number of targets including DNA and other macromolecules. It is tempting to speculate that the predictable (i.e., being achieved in the great majority of all treated patients) curative effect might be due to, in addition to all of the lesions inflicted by this reuse of available therapeutic agents, an unintended effect on some as yet unknown component of chaotic behavior. For example, this latter effect might have altered a balance optimum point within a physiological stress response mechanism and by this means have mediated an immune attack that eliminated a few dormant and treatment-resistant leukemia cells, and thus also the risk for a deadly relapse. In this context, it is relevant to note that a recent perspective article on the mechanisms mediating cancer chemotherapy’s curative effect argues that research into non-cytotoxic effects may bring insights into novel potent therapies (14). Arguably, there was an element of tinkering also in the discovery of ICB, since it came in the wake of less successful attempts to target immunoregulatory molecules. As a therapy it represents the currently dominating targeting strategy which is well-suited for a complicated problem, and this may explain the therapy’s unpredictable and sometimes complete lack of clinical effect (54). Thus, tinkering may overlap with what we refer to as chaos theory; when a tinkering attempt is successful it is sometimes because it happens to interfere with a complex and dynamic normal physiologic defense mechanism.

In common language the word tinkering often refers to a non-skilled person making a qualified guess on how to solve a problem in a trial-and-error manner, using only whatever tools and materials there is at hand. If you have any doubt whatsoever that human scientific endeavors within a foreseeable future will make us master cancer, then such tinkering should be judged to be a reasonable supplementary research strategy. If pediatricians made the qualified guess that cytotoxic agents can cure leukemia and they did find the necessary tools in their available arsenal, and if the qualified but previously disputed hypothesis that regulation of adaptive immunity can cure malignant melanoma eventually succeeded to give us the checkpoint blockade tools; then, can we suggest similar qualified guesses worthy of testing with tinkering? One candidate is to focus on how to adjust the balance points within the normal physiology of defense (against, for example, cancer) which have been defined by evolution, and thus go against evolution and serve the interest of the cancer patient instead of the species. If we are smart enough to understand how smart evolution has been (to paraphrase Frans de Waal), then we should advocate this candidate. Another candidate may be based on the observation that many cancer forms and therapies are strongly associated with the composition of the intestinal microbiome, suggesting that trials with fecal or microbe-specific transplantations are worthwhile (55, 56). The high burden of cancer in obese people, when taken together with emerging evidence of cancer reduction in people taking appetite-suppressing metabolism modifiers, indicates another area worth tinkering with.

## Conclusions

In summary, knowledge on complexity is emerging from many disciplines which suggests that the present cancer research strategy needs to be diversified. The currently dominating experimental cancer research strategy is incomplete, while not incorrect. It must be supplemented with a strategy type based on chaos theory and tinkering.
